# Oxygen conserving mitochondrial adaptations in the skeletal muscles of breath hold divers

**DOI:** 10.1371/journal.pone.0201401

**Published:** 2018-09-19

**Authors:** Thomas Kjeld, Nis Stride, Anders Gudiksen, Egon Godthaab Hansen, Henrik Christian Arendrup, Peter Frederik Horstmann, Bo Zerahn, Lars Thorbjørn Jensen, Nikolai Nordsborg, Jacob Bejder, Jens Frey Halling

**Affiliations:** 1 Department of Anesthesiology, Herlev Hospital, Herlev, University of Copenhagen, Denmark; 2 Department of Cardiology, Rigshospitalet, Copenhagen, University of Copenhagen, Denmark; 3 Department of Biology, University of Copenhagen, Copenhagen, Denmark; 4 Department of Cardiothoracic Surgery, Rigshospitalet, Copenhagen, University of Copenhagen, Denmark; 5 Department of Ortopedic Surgery, Hillerød Hospital, Hillerød, University of Copenhagen, Denmark; 6 Department of Clinical Physiology and Nuclear Medicine, Herlev Hospital, Herlev, University of Copenhagen, Denmark; 7 Department of Nutrition, Exercise and Sport (NEXS), Copenhagen, University of Copenhagen, Denmark; University of California Los Angeles, UNITED STATES

## Abstract

**Background:**

The performance of elite breath hold divers (BHD) includes static breath hold for more than 11 minutes, swimming as far as 300 m, or going below 250 m in depth, all on a single breath of air. Diving mammals are adapted to sustain oxidative metabolism in hypoxic conditions through several metabolic adaptations, including improved capacity for oxygen transport and mitochondrial oxidative phosphorylation in skeletal muscle. It was hypothesized that similar adaptations characterized human BHD. Hence, the purpose of this study was to examine the capacity for oxidative metabolism in skeletal muscle of BHD compared to matched controls.

**Methods:**

Biopsies were obtained from the lateral vastus of the femoral muscle from 8 Danish BHD and 8 non-diving controls (Judo athletes) matched for morphometry and whole body VO_2_max. High resolution respirometry was used to determine mitochondrial respiratory capacity and leak respiration with simultaneous measurement of mitochondrial H_2_O_2_ emission. Maximal citrate synthase (CS) and 3-hydroxyacyl CoA dehydrogenase (HAD) activity were measured in muscle tissue homogenates. Western Blotting was used to determine protein contents of respiratory complex I-V subunits and myoglobin in muscle tissue lysates.

**Results:**

Muscle biopsies of BHD revealed lower mitochondrial leak respiration and electron transfer system (ETS) capacity and higher H_2_O_2_ emission during leak respiration than controls, with no differences in enzyme activities (CS and HAD) or protein content of mitochondrial complex subunits myoglobin, myosin heavy chain isoforms, markers of glucose metabolism and antioxidant enzymes.

**Conclusion:**

We demonstrated for the first time in humans, that the skeletal muscles of BHD are characterized by lower mitochondrial oxygen consumption both during low leak and high (ETS) respiration than matched controls. This supports previous observations of diving mammals demonstrating a lower aerobic mitochondrial capacity of the skeletal muscles as an oxygen conserving adaptation during prolonged dives.

## Introduction

Diving mammals are adapted to sustained aerobic metabolism under hypoxic conditions, and relative to comparable terrestrial mammals, skeletal muscles of pinnipeds (seals and sea lions) have higher volume density of mitochondria [Vv_(mt)_] and correspondingly higher citrate synthase (CS) activity, higher beta-hydroxyacyl CoA dehydrogenase (HAD) activity and thus a higher capacity for fatty acid catabolism for aerobic ATP production [1]. Oxygen storage and diffusion capacity is higher due to an increased myoglobin concentration and dependency on blood-borne oxygen and metabolites is lower as indicated by a decreased capillary density [1]. These adaptations may be speculated to result in higher mitochondrial respiratory capacity. However, the oxidative phosphorylation (OXPHOS) capacity of the northern elephant seal (NES) muscle is generally lower than in humans [2], but the difference is leak respiration in NES is less with lipid-based substrates (palmitoylcarnitine + malate) than with pyruvate. This supports that a relatively low mitochondrial capacity with a preference for lipid oxidation contributes to an improved diving performance. Together, these metabolic features expand the animals dive capacity, while relying primarily on oxygen stored in blood and muscle. Hence, these pinnipeds exhibit a distinct metabolic adaptation to their frequent long dives that may serve to preserve oxygen for aerobic metabolism in heart, liver, kidneys, gastrointestinal organs and skeletal muscle [1,3,4]. Mitochondrial biogenesis in human skeletal muscle is highly plastic with one suggested regulator being tissue hypoxia [5] and it could therefore be hypothesized that the same adaptive mechanisms are present in human elite breath hold divers (BHD), who endure breath holds for more than 11 min, swimming submerged as far as 300 m or going below 250 m in depth, all on a single breath of air (www.aida.international.org).

Like other animals, humans possess a diving response that is initiated during apnea and augmented with facial immersion in cold water [6–8]. The diving response includes peripheral vasoconstriction, a reduced cardiac output (CO), bradycardia [9] and low muscle oxygenation, whereas cerebral perfusion is augmented as hypercapnia develops [10–12]. At the end of a dry breath hold, BHD demonstrates an augmented diving response, including attenuated post-apnoea acidosis, decreased oxidative stress, and probably critical for their performance an extreme tolerance for hypoxia and hypercapnia [7,8]. Also, plasma erythropoietin increases, while BHD tolerate a ~33% reduction in frontal lobe oxygenation and a ~50% reduction in muscle oxygenation [11,12]. However, it is currently unknown whether skeletal muscle of trained human free divers exhibits mitochondrial adaptations that may contribute to the enhanced diving response that allows the extreme performances by BHD.

Hence, the purpose of this study was to examine the capacity for oxidative metabolism in skeletal muscle of BHD compared to matched controls. We postulated that, compared to matched non-diving controls, skeletal muscle from human BHD would be characterized by 1) enhanced oxygen storage and diffusion capacity, 2) elevated mitochondrial density as estimated by CS activity, 3) greater capacity for fatty-acid oxidation and mitochondrial oxidative phosphorylation and 4) reduced mitochondrial emission of reactive oxygen species (ROS).

## Methods

This research involving human participants have been approved by the Regional Ethics Committee of Copenhagen (H-1-2013-060). All clinical investigations have been conducted according to the principles expressed in the Declaration of Helsinki. Informed consent, written and oral, have been obtained from the participants.

16 healthy male non-smoking subjects participated in the study. 8 subjects were divers (age 42 ± 8 years) and for comparison were chosen 8 judo athletes matched for morphometric variables (age, height, weight, body mass) and whole-body aerobic capacity (VO_2_max).

All free divers ranked among national top 10, three of the participating free divers ranked among World top 10 and one was a 2016 outdoor free-diving World champion, while one reached third place at the same Championship (no limit depth competition), and one was a World record holder.

### VO_2_ max and dual-energy X-ray absorptiometry scan

Subjects completed a standardized warm-up followed by an incremental cycling test starting at a workload of 150 W and increasing 25 W every minute until voluntary exhaustion. Pulmonary O_2_ and CO_2_ concentrations in the expired gas were continuously measured breath-by-breath (Quark, Cosmed, Rome, Italy) during the test. The highest recorded 30 s average oxygen uptake (VO_2_) during the test was defined as VO_2_max. For recognition of true VO_2_max three of five criterions had to be met: individual perception of exhaustion, respiratory exchange ratio > 1.15, plateau of VO_2_ curve, heart rate approaching age-predicted maximum and inability to maintain a pedaling frequency above 70 rpm. A dual-energy X-ray absorptiometry scan (Lunar iDXA; Lunar, Madison, WI, USA) was performed to assess body composition (Table 1).

**Table 1:** Subject characteristics.

	Controls	Divers
No. subjects	8 males	8 males
Age (years)	36 ± 11	42 ± 8
Height (cm)	183 ± 4	183 ± 6
Weight (kg)	82 ± 6	79 ± 6
Body Surface area (Mosteller, m^2^)	2.0 ± 0.1	2.0 ± 0.1
Body Mass Index (kg/m^2^)	23.6 ± 1.2	24.3 ± 1.5
Fat mass %	15.4 ± 6.2	20.2 ± 4.2
Bone Mineral Content (kg)	3.7 ± 0.4	3.1 ± 0.2 *
Fat (kg)	12.7 ± 6.0	15.9 ± 3.7
Fat free mass (kg)	66.3 ± 3.9	60.9 ± 5.2 *
Bone Mineral Density (kg/m^2^)	1.4 ± 0.1	1.3 ± 0.1 *
T/Z-score	2.3 ± 0.8	0.6 ± 0.8 *
Maximal oxygen uptake (ml O2/min)	4018 ± 398	3676 ± 503
Capillary Hemoglobin (g/dl)	15.3 ± 0.8	15.2 ± 0.8
Venous Hemoglobin (calculated [17], g/dl)	14.7 ± 0.7	14.6 ± 0.7
Static personal best (seconds)	N/A	403 ± 62
Dynamic pool personal best (meters)	N/A	163 ± 36
Dynamic pool no fins personal best (meters)	N/A	133± 37

Basic morphometric data. Values are mean ± SD.

* P < 0.05

### Muscle biopsies

Muscle biopsies were obtained in local anesthesia with lidocaine (5%) from the lateral vastus of the femoral muscle a.m. Bergstroem [13]. One part of the biopsy was instantaneously submerged into ice cold buffer solution, ‘BIOPS’ [14] containing buffer (50mM K-MES, 7.23mM K_2_EGTA, 2.77mM CaK_2_EGTA, 20mM imidazole, 20mM taurine, 5.7mM ATP, 14.3mM phosphocreatine and 6.56mM MgCl_2_, pH 7.1) until preparation of permeabilized fiber bundles (PmFB). Fiber bundles weighing 3-5mg were teased with fine forceps and permeabilized in 30 μg/mL saponin in BIOPS for 30 min at 5°C. PmFBs were then washed in ice-cold mitochondrial respiration medium (MiR05: 0.5mM EGTA, 3mM MgCl_2_, 60mM lactobionate, 20mM taurine, 10mM KH_2_PO_4_, 20mM HEPES, 110mM D-sucrose, 1g/L BSA) for 30 min before analysis. After each respiration protocol, PmFBs were extracted from the respiration chamber and weighed after vacuum-drying. Another part of the biopsy was snap-frozen in liquid N_2_ and stored at −80°C for later assay of enzyme activities and protein content.

### Mitochondrial respiration and H_2_O_2_ emission

High-resolution O_2_ consumption was measured in MiR05 in the Oxygraph-2k system (Oroboros Instruments, Innsbruck, Austria). Respiration measurements were performed in duplicate at 37°C with [O2] at ∼400–180μM. Briefly, Complex I supported leak respiration was measured after addition of 5mM pyruvate, 10mM glutamate and 2mM malate. Maximal Complex I supported oxidative phosphorylation (OXPHOS) capacity was measured after addition of ADP (4mM). Complex I+II supported OXPHOS capacity was measured after succinate addition (10mM). Electron transfer system (ETS) capacity through Complex I+II was measured after sequential additions of 0.5μM FCCP. Finally, 1μM rotenone was added to inhibit Complex I.

Emission of H_2_O_2_ (defined as H_2_O_2_ escaping the mitochondrial matrix) was measured simultaneously with O_2_ consumption using the O2k-Fluo LED2-Module (Oroboros Instruments, Innsbruck, Austria). Briefly, Horseradish peroxidase (4U/mL) and Amplex Red (10μM) was added and the H_2_O_2_ mediated conversion of Amplex Red to resorufin was tracked by excitation/emission at 565/600nm. Superoxide dismutase (30U/mL) was added to ensure conversion of superoxide to H_2_O_2_.

### Immunoblotting

Muscle lysates were made from freeze-dried human muscle biopsies dissected free of connective tissue, blood and visible fat. The tissue was homogenized in a ratio of 1:80 in lysis buffer (10% glycerol, 20 mM Na-pyrophosphate, 150 mM NaCl, 50 mM HEPES, 1% NP-40, 20 mM β-glycerophosphate, 10 mM NaF, 1 mM EDTA, 1 mM EGTA, 20 μg/ml aprotinin, 10 μg/ml leupeptin, 2 mM Na_3_VO_4_, 3 mM benzamidine, and adjusted to pH 7.4) using a Tissue Lyser II (Qiagen, Germany). Protein concentrations were determined using the bicinchoninic acid method (Thermo Fischer Scientific, USA) and were adjusted with sample buffer to a concentration of 1μg/μl for each sample. Specific protein content of OXPHOS (Abcam/ab110413) GLUT4 (Thermo-Fisher/ PA1-1065), Hexokinase II (HKII) (Cell Signaling/#2867), Catalase (Santa Cruz/50508), SOD2 (Abcam/74231), pyruvate dehydrogenase subunit E1α (PDH-E1α) (Prof. Graham Hardie, University of Dundee, Dundee, Scotland) and myoglobin (Abcam/ab77232) were determined by SDS-PAGE using hand casted gels and western blotting. PVDF membranes were incubated in primary antibody overnight at 4°C. Species-specific horseradish peroxidase conjugated immunoglobulin secondary antibodies (DAKO, Denmark) were used for incubation the following day. Protein bands were subsequently visualized using an ImageQuant LAS 4000 imaging system and quantified with ImageQuant TL 8.1 software (GE Healthcare, Germany).

### Myosin heavy chain determination

Myosin heavy chain (MHC) composition of the *m*. *vastus lateralis* muscle was determined from muscle homogenate using gel electrophoresis as described previously [15]. In brief, muscle homogenates were diluted with 6×Laemmli sample-buffer (7 ml 0.5 M Tris-base, 3 ml glycerol, 0.93 g DTT, 1 g SDS and 1.2 mg bromophenol blue) and 100% glycerol (50/50). A total of 1 μg of protein was separated on 8% self-cast stain free gels (49:1 acrylmid: bis-acrylmid, 30% glycerol, 200 mM Tris-base, 0.4% SDS, 0.1% APS and 0.1 M glycine) containing 0.5% 2,2,2 Trichloroethanol [16] for 16 h at 140 V on ice. MHC protein bands were visualized by ultraviolet activation of the stain free gel (ChemiDoc MP Imaging System) and were quantified densitometrically using imaging software (Image Lab v. 4.0, Bio-Rad Laboratories, Hercules, CA, US).

### Enzyme activities

Maximal CS activity was measured in triplicates spectrophotometrically following the protocol of the manufacturer (CS0720, Sigma Aldrich, Germany). 3-hydroxyacyl-CoA dehydrogenase activity was determined in triplicates kinetically by measuring fluorescence (excitation 355nm/emission 460nm; Fluoroscan, Thermo Scientific, USA)) after addition of acetoacetyl CoA as previously described (Lowry OH, Passonneau JV. A Flexible System of Enzymatic analysis. London: Academic, 1972). CS and HAD activities were normalized to muscle weight.

### Statistics

Data were analyzed using a One-way ANOVA. Holm Sidak method was used to evaluate differences between the two groups of subjects. A P-value < 0.05 was considered statistically significant.

## Results

### VO_2_ max and dual-energy X-ray absorptiometry scan

The divers were characterized by ~16% lower bone mineral content (divers 3.1 ± 0.2 kg vs. controls 3.7 ± 0.4 kg, P <0.05) and ~ 9% lower fat free mass (divers 60.9 ± 5.2 vs. controls 66.3 ± 3.9, P<0.05) than controls (Table 1).

### Respirometry

**Fig 1:**
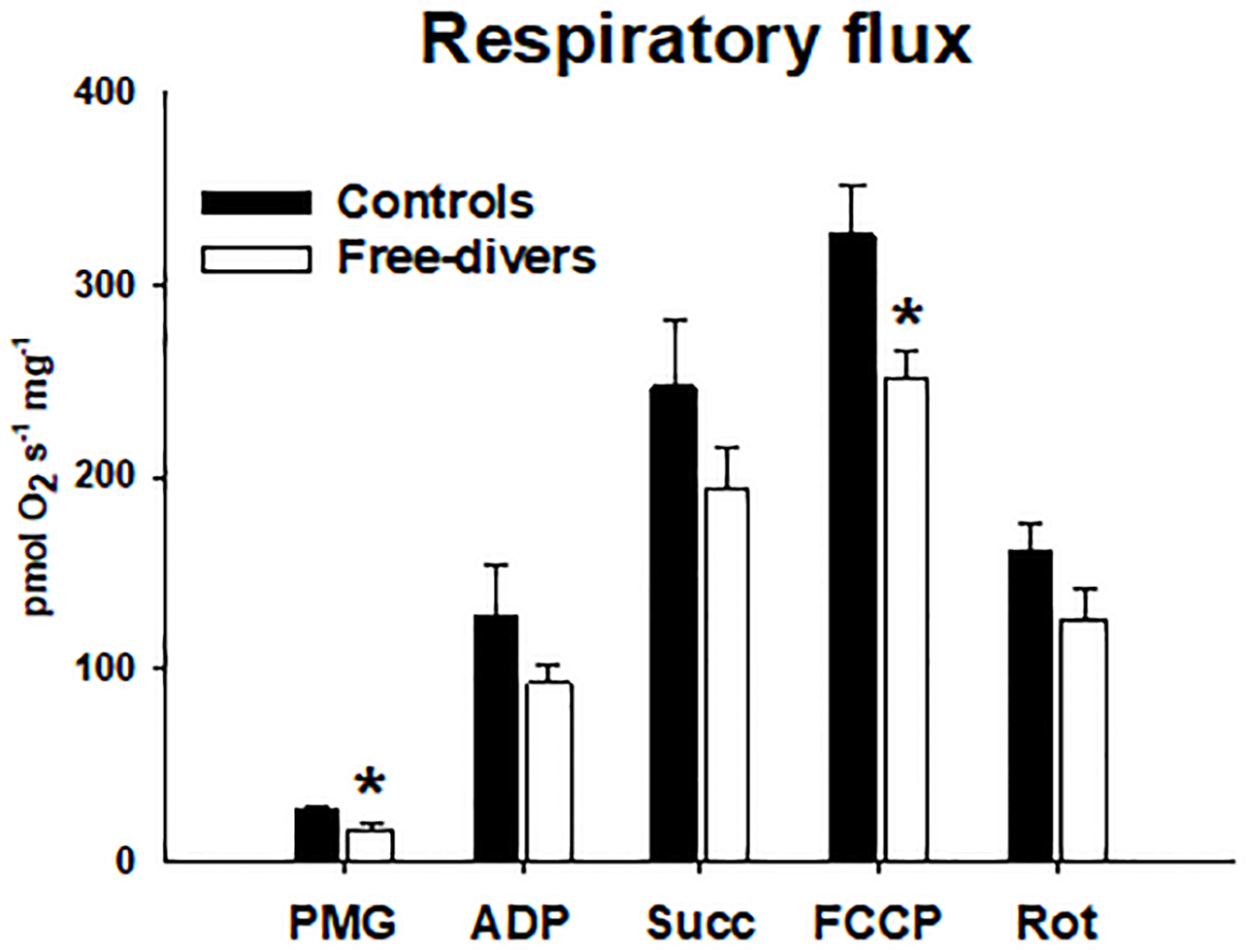
Respiratory flux. PMG (Pyruvate, Malate, Glutamate) divers 16.6 ± 3.3 vs. controls 26.8 ± 3.2, P<0.05. ADP Adenosine Di-Phosphate) divers 92.9 ± 10.3 vs. controls 127.2 ± 26.5. Succ (Succinate) divers 193.8 ± 21.1 vs. controls 247.0 ± 34.7, FCCP (Carbonyl cyanide-p-trifluoromethoxyphenylhydrazone) divers 251.1 ± 14.4 vs. controls 326.2 ± 25.5, P = 0.006. Rot (Rotenone) divers 126.5 ± 16.1 vs. controls 161.5 ± 15.4. * P <0.05.

Complex I leak capacity was ~22% lower in divers than in controls in divers (16.6 ± 3.3 vs. controls 26.8 ± 3.2, P<0.05), and similarly maximal ETS Complex I+II capacity were ~ 23% lower in divers than in controls, (divers 251.1 ± 14.4 vs. controls 326.2 ± 25.5, P = 0.006) (Fig 1).

**Fig 2:**
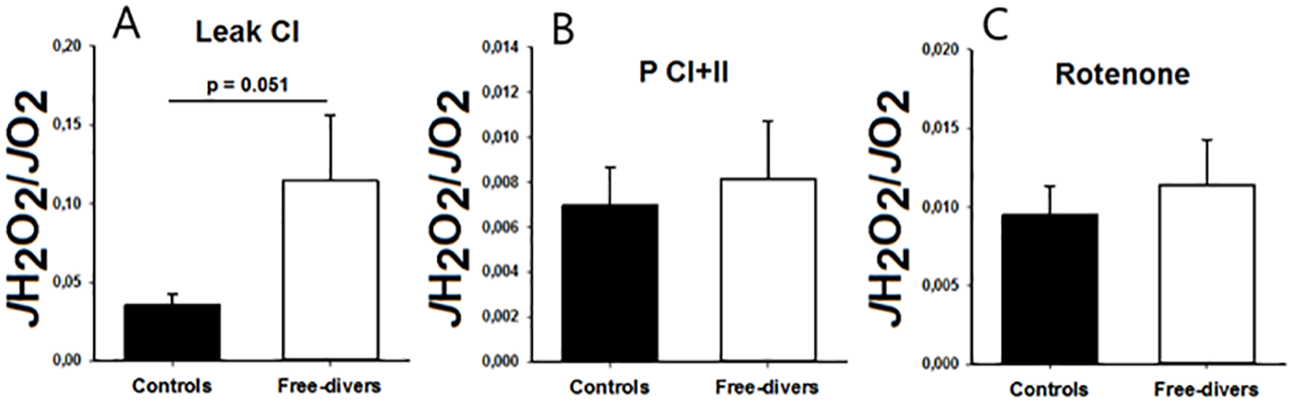
A-C. JH_2_O_2_/JO_2_ (Mitochondrial respiratory function / Hydrogen Peroxide Emission. (A) Leak Complex I Divers 0.12 ± 0.05 vs. Controls 0.04 ± 0.007, P = 0.051. (B) Complex I+II Divers 0.008 ± 0.003 vs. Controls 0.007 ± 0.002. (C) Rotenone Divers 0.011 ± 0.003 vs. Controls 0.009 ± 0.007.

Results for mitochondrial respiration and H_2_O_2_ emission were not significantly different between the two groups, however, during Complex I-linked leak respiration (without ADP present) the mitochondrial emission of H2O2 normalized to O2 consumption tended to be higher (p = 0.051) in permeabilized muscle fibers from free-divers compared with controls (Fig 2).

### OXPHOS, myoglobin and CS and HAD activities

OXPHOS protein content, Myoglobin protein content, and CS and HAD activities were not significantly different between the two groups:

Specific content of OXPHOS CI (NDUFB8 = NADH dehydrogenase [ubiquinone] 1 beta subcomplex subunit 8) in divers 1.0 ± 0.1 and in controls 1.0 ± 0.1, CII (SDHB = Succinate dehydrogenase [ubiquinone] iron-sulfur subunit) in divers 1.0 ± 0.1 and in controls 1.0 ± 0.1, CIII (UQCRC2 = ubiquinol-cytochrome c reductase complex) in divers 0.9 ± 0.1 and in controls 1.0 ± 0.1, CIV (MTCOI = mitochondrial cytochrome oxidase I) in divers 0.9 ± 0.1 and in controls 1.0 ± 0.2, and CV (ATP5A = ATP synthase subunit alpha) in divers 0.9 ± 0.1 and in controls 1.0 ± 0.1.

Specific content of myoglobin in divers 1.00 ± 0.03 and in controls 1.00 ± 0.03.

**Fig 3:**
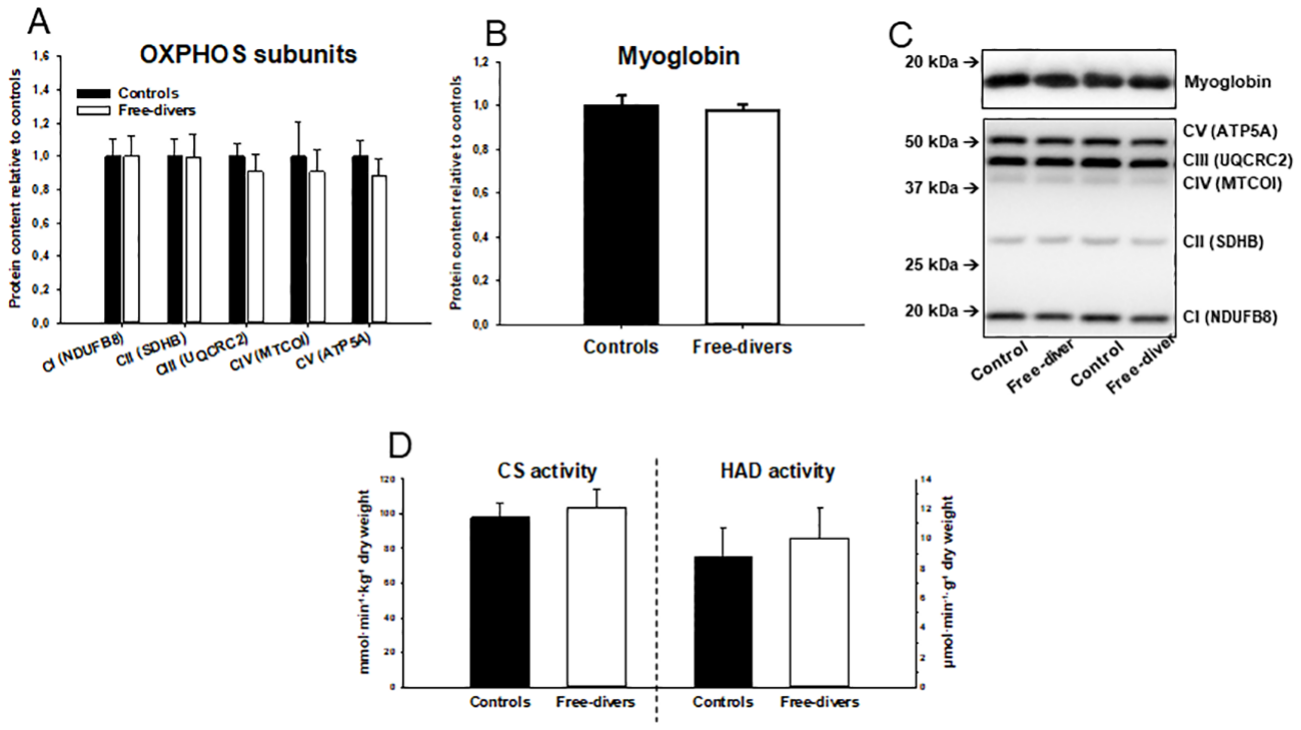
A-D. OXPHOS, Myoglobin and HAD activities. (A) Specific content of OXPHOS CI (NDUFB8 = NADH dehydrogenase [ubiquinone] 1 beta subcomplex subunit 8) divers 1.0 ± 0.1 vs controls 1.0 ± 0.1, CII (SDHB = Succinate dehydrogenase [ubiquinone] iron-sulfur subunit) divers 1.0 ± 0.1 vs controls 1.0 ± 0.1, CIII (UQCRC2 = ubiquinol-cytochrome c reductase complex) divers 0.9 ± 0.1 vs controls 1.0 ± 0.1, CIV (MTCOI = mitochondrial cytochrome oxidase I) divers 0.9 ± 0.1 vs controls 1.0 ± 0.2, CV (ATP5A = ATP synthase subunit alpha) divers 0.9 ± 0.1 vs controls 1.0 ± 0.1. (B) Specific content of myoglobin divers 1.00 ± 0.03 vs. controls 1.00 ± 0.03. (C) SDS-page and western blot for determination of myoglobin. (D) Maximal CS (citrate synthase) activity divers 103.9 ± 10.0 vs. controls 98,0 ± 8.4 and HAD (3-hydroxyacyl CoA dehydrogenase) activity divers 10.0 ± 2.0 vs. controls 8.8 ± 2.0.

Maximal CS (citrate synthase) activity in divers 103.9 ± 10.0 and in controls 98,0 ± 8.4 and HAD (3-hydroxyacyl CoA dehydrogenase) activity in divers 10.0 ± 2.0 and in controls 8.8 ± 2.0 (Fig 3).

### Glucose metabolism and antioxidant enzymes

**Fig 4:**
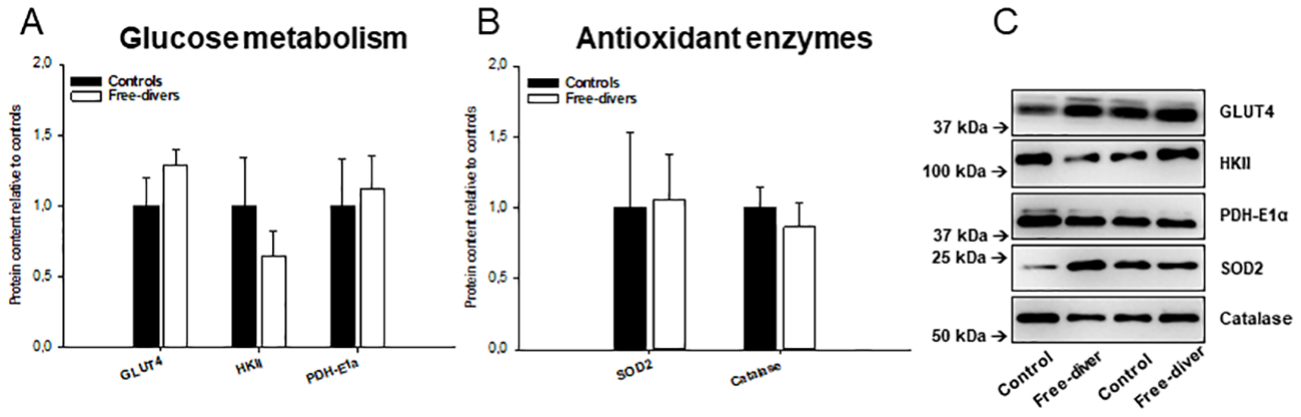
A-C. Glucose metabolism and antioxidant enzymes. (A) Glucose metabolism: GLUT4 (divers 1.11 ± 0.09 vs. controls 0.86 ± 0.17), HKII (divers 0.83 ± 0.23 vs. controls 1.30 ± 0.44) and PDH-E1α (divers 2.47 ± 0.49 vs. controls 2.20 ± 0.72). (B) Antioxidant enzymes: protein content of superoxide dismutase (SOD2) (divers 2.49 ± 0.76 vs. controls 2.35 ± 1.26) and Catalase (divers 0.91 ± 0.18 vs. controls 1.05 ± 0.15). (C) SDS-page and western blot for determination of proteins mentioned above.

Protein contents were measured as markers of the capacity for glucose metabolism through glycolysis and results were similar in free divers and controls:

GLUT4 (divers 1.11 ± 0.09 vs. controls 0.86 ± 0.17), HKII (divers 0.83 ± 0.23 vs. controls 1.30 ± 0.44) and PDH-E1α (divers 2.47 ± 0.49 vs. controls 2.20 ± 0.72) were similar in divers and controls. In addition, protein content of superoxide dismutase (SOD2) (divers 2.49 ± 0.76 vs. controls 2.35 ± 1.26) and Catalase (divers 0.91 ± 0.18 vs. controls 1.05 ± 0.15) were measured as markers of mitochondrial and cytosolic antioxidant capacity, respectively. However, no difference in antioxidant capacity was observed between free divers and controls (Fig 4).

### Myosin heavy chain

The relative distribution of MHCI and MHCII isoforms in m. vastus lateralis were similar in free divers (60±11% and 40±11%, respectively) and controls (58±15% and 42±15%, respectively).

## Discussion

The main findings of the present study are that permeabilized skeletal muscle fibers from competitive free divers had lower capacity for mitochondrial oxygen consumption both during non-phosphorylating (leak) and uncoupled (ETS) respiration than muscle fibers from matched controls (Fig 1). Free divers also tended to display higher ROS emission during leak respiration (Fig 2). This was observed independently of differences in protein content of OXPHOS subunits as well as CS and HAD activities (Fig 3), suggesting that human competitive free divers are characterized by lower intrinsic mitochondrial function. This has to our knowledge not previously been described.

BHD are characterized by a significant diving response, attenuated post-apnoea acidosis, oxidative stress and, a reduced sensitivity to hypoxia and hypercapnia [7,8,10], beyond the limits of consciousness and organ damage in non-adapted humans. Weeks of apnea training in untrained subjects can improve the diving response, with an increased apnea time and a pronounced bradycardia [8]. The present findings indicate that adaptations in favor of reduced skeletal muscle mitochondrial oxygen consumption contributes to the augmented diving response with reduced sensitivity to post-apnoea acidosis and oxidative stress as observed in BHD.

Chicco et al demonstrated that, permeabilized muscle fibers of adult northern elephant seals (NES) exhibit lower mitochondrial oxidative capacity than non-diving humans. However, the ratio of lipid versus pyruvate/malate supported respiration was comparably higher in NES than in human muscle [2], suggesting higher relative capacity for fat oxidation in diving seals. This was interpreted as an adaptation allowing lower skeletal muscle oxygen extraction during dives, which would increase the dive limit by saving the limited oxygen stores for the most central organs [2]. Adult seals also seem to have a higher relative capacity for leak respiration than pups and juvenile seals as well as humans, which was hypothesized to facilitate thermoregulation during deep dives where temperatures are highly variable [2]. The present finding that mitochondrial respiratory capacity was lower in free divers than controls (Fig 1), independently of differences in markers of mitochondrial content, CS activity, and protein content of respiratory complex subunits (Fig 3), suggests that intrinsic functional characteristics of mitochondrial respiration contribute to save oxygen and thus improve diving performance in human free divers. Summarized the findings in our study indicates that adaptations in favor of reduced skeletal muscle mitochondrial oxygen consumption contributes to the augmented diving response with reduced sensitivity to post-apnoea acidosis and oxidative stress as observed in elite breath hold divers.

The peripheral vasoconstriction induced as part of the diving reflex greatly reduces convective oxygen delivery. Therefore, the capacity for myoglobin-mediated oxygen diffusion into skeletal muscle during hypoxia may be essential for maintaining muscle oxygenation during long dives. Accordingly, it has been shown that skeletal muscles of adult Weddell seals possess 8-13-fold higher myoglobin concentration compared with dog hindlimb muscle [1]. Therefore, we hypothesized that higher skeletal muscle myoglobin content would facilitate improved oxygen storage and diffusion capacity in BHD compared with controls. However, the similarity in myoglobin content observed in the BHD and controls (Fig 3), suggests that myoglobin-mediated peripheral oxygen storage and diffusion capacity does not contribute to diving performance in humans. Interestingly, modelling aerobic dive limits of seals at different myoglobin concentrations showed that increased myoglobin content may not necessarily increase the dive capacity, as convective and diffusive oxygen transport is matched and optimized for diving [18]. Monitoring the ontogeny of Weddell seals revealed that adult seals unexpectedly have lower muscular myoglobin concentration than juvenile seals, which are not yet fully matured and adapted to long dives [4]. Together, experimental evidence from diving mammals suggests that adaptations leading to reduced skeletal muscle oxygen storage and diffusion capacity is associated with improved diving performance. However, the present results suggest that changes in myoglobin content do not contribute to diving performance in competitive human free divers.

An interesting previous observation is that although lipid oxidation is less efficient in energetic terms, OXPHOS capacity of NES muscle with lipids is ~ 75% of that in human muscle, whereas pyruvate/malate/succinate-linked OXPHOS capacity is only ~ 43% of that in human muscle. Together with other similar observations, this has led to the notion, that diving mammals predominantly use lipid metabolism for muscle ATP production [3,4]. It has been shown that myoglobin can bind and transport fatty acids and thus regulate myocellular substrate utilization [19]. This provides a possible link between the paradoxical apparent preference for lipid oxidation in diving mammals: various species of diving seals have comparably high myoglobin levels [1,20,21]. However, in the present study there was no evidence pointing towards a preference for fatty acid oxidation in skeletal muscle from BHD, as indicated by myoglobin content and HAD activity.

During a long breath hold or dive, skeletal muscles become hypoxic followed by reperfusion with the next breath of air. Tissue hypoxia, or in its extreme, ischemia, is well known to cause harmful oxidative damage, due to bursts in ROS emission from mitochondria [22]. Interestingly, although diving seals habitually undergo repeated ischemia/reperfusion events when diving, they do not display indications of oxidative tissue damage [3,23,24]. Because previous studies have demonstrated that skeletal muscle mitochondria of diving animals displayed marked adaptations, they might be associated with reduced ROS emission, which could explain the apparent protection against oxidative damage. In our study we observed that absolute levels of H_2_O_2_ emission were similar in controls and BHD (data not shown). Surprisingly, when normalizing for oxygen consumption, H_2_O_2_ emission tended to be higher in free divers than controls during leak respiration (P = 0.051, Fig 2). Mitochondrial ROS emission is most pronounced during non-phosphorylating conditions with high substrate supply, because electron transport becomes blocked when protons are not allowed to pass through the ATP synthase [25]. The higher rate of mitochondrial H_2_O_2_ emission in free divers was only apparent under non-phosphorylating conditions (Fig 2), which is consistent with the finding that free divers also exhibited lower capacity for leak respiration (Fig 1). Therefore, the observed reduced capacity for mitochondrial oxygen consumption, that may contribute to improve diving performance in BHD, simultaneously leads to increased ROS emission. Animals that routinely face high changes in oxygen availability and/or consumption seem to show a general strategy to prevent oxidative damage by having either appropriate high constitutive antioxidant defenses and/or the ability to undergo arrested states, where depressed metabolic rates minimize the oxidative challenge [26]. Hence, to avoid exposure of tissues to changing high oxygen levels, and therefore to avoid an oxidative stress condition related to antioxidant consumption and increased ROS generation, diving mammals possess constitutive high levels of antioxidants in tissues. Ringed seal muscle, can be induced in vitro to generate ROS [24]. This suggests that the protective mechanisms of living seals depend on O(2)(.-) production, (similar to the protective effect of experimental preconditioning) [23]. Hence, the present study of BHD confirms the findings of increased ROS emission in skeletal muscles as observed in diving mammals. This has to our knowledge never previously been demonstrated.

Diving mammals have, in general, higher antioxidant capacities compared to non-diving mammals [26], and in rodents this has been shown in muskrats (Ondatra zibethicus), a semi-aquatic rodent, which exhibits a higher superoxide dismutase activity in almost all tissues compared to beaver (Castor fiber) and nutria (Myocastor coypus) [27]. Bulmer et al. demonstrated that trained free divers have increased erythrocyte superoxide dismutase activity during apnea, but only a small difference in their antioxidant and oxidative stress responses compared with controls [28]. In our study, no significant differences in antioxidant enzyme content or activity were found in the skeletal muscle between the two groups.

Expression of GLUT4, which facilities uptake of circulating glucose into skeletal muscle, and expression of the glycolytic enzyme hexokinase II, playing important role in glucose metabolism, were not different between the two groups (Fig 4). In addition, PDH-E1α content was similar in BHD and controls (Fig 4), together suggesting that the capacity for glycolysis and linking glycolytic and mitochondrial metabolism is not altered in BHD.

Phosphofructokinase also play an important role in (muscle) glucose metabolism and fructose-driven glycolytic respiration in naked mole-rat tissues were recently discovered in vitro to avoid feedback inhibition of glycolysis via phosphofructokinase, a possible mechanism concluded could be harnessed in minimizing hypoxic damage in human disease [29], However, our study demonstrates an actual human adaption to hypoxia.

The observation that the BHD in the present study had lower fat free mass than controls, indicates a lower oxygen demand per kilogram body weight in the divers than the control. Moreover, the lower BMD in the divers than the controls in the current study is in line with studies, demonstrating that BMD of divers and swimmers tends to be lower than in non-diving controls [30]. This may be caused by the weight-supported environment in the water, exerting an effect that reduces BMD [31] proportionately to the time spent in the water [32].

In conclusion, the present study demonstrates for the first time, that the skeletal muscles of BHD are characterized by lower mitochondrial oxygen consumption both during low leak and high (ETS) respiration than matched controls. This supports previous observations of diving mammals demonstrating a lower aerobic mitochondrial capacity of the skeletal muscles, reflecting their energy-conserving modes of locomotion as an oxygen conserving adaption during prolonged dives.

## Supporting information

S1 Table (XLSX)

S2 Table (XLSX)

S3 Table (XLSX)

S4 Table (XLSX)
